# 

**Published:** 1897-03-20

**Authors:** 


### EARLY EDITION.

**Notices of Births, Deaths, and Marriages** are inserted in this column at a charge of 2s. 6d. for an announcement not exceeding four lines.

### NOTICE TO CORRESPONDENTS.

All MS., Letters, Books for Review, and other matters intended for the Editor should be addressed The Editor, "The Hospital," The Hospital Building, 28 & 29, Southampton Street, Strand, W.C.

The Editor cannot undertake to return rejected MS., even when accompanied by stamped directed envelope.

**Notice to Advertisers and Subscribers.**

All Advertisements, Orders for Copies of The Hospital, and Business Communications should be addressed to **THE MANAGER, (Not to The Editor),** The Hospital, The Hospital Building, 28 & 29, Southampton Street, Strand, London, W.C.

The Cost of Subscription, post free, is as follows:—Per week, 2½d.; per quarter, 2s. 8d.; per half-year, 5s. 3d.; per annum, 10s. 6d. Foreign:—Per annum, 15s. 2d.; payable, in all cases, in advance.

**NOTICE.—Vol. XX. of "The Hospital" (April to September, 1896), in handsome cloth binding, is now on sale at the Office of this Journal, price 7s. 6d. Cases for binding the Numbers contained in this Volume, 1s. 6d. Index to Vol. XX., 2½d., post free.**

**The Monthly Part for April will be ready on April 1st. Price 6d., by post 8d.**

The Howard Association is issuing leaflets, entitled "Morocco Prisons in 1897," from which we are glad to learn that some of the terrible practices reported some years ago have ceased to prevail, and that the general condition of prison life shows much improvement. This is attributed to European action and opinion, to which, doubtless, the English nation can lay claim to having largely contributed, and the Howard Association not a little.

### The Student of Medicine.

### APPOINTMENTS.

T. S. Byass, M.R.C.S., L.R.C.P.Lond., appointed District Medical Officer to the Wallingford Union, *vice* W. C. Byass, M.R.C.S.Eng., L.S.A., resigned. H. Caiger, M.B.Lond., F.R.C.S.Eng., appointed Railway Medical Officer, Burghersdorp, Cape Colony. Mr. E. L. Davey, appointed Medical Officer of Health for the Walmer Urban District. Dr. Drewitt, appointed Second Assistant Medical Officer to the Chelsea Union. L. A. Dunn, F.R.C.S. M.S., appointed Consulting Surgeon to the St. Mary's Children's Hospital, Plaistow, E. R. H. Fagge, M.R.C.S.Eng., L.R.C.P.Lond., appointed House Surgeon to the Leicester Infirmary. W. W. Farnfield, M.R.C.S., L.R.C.P., appointed Resident Medical Officer to St. Mary's Children's Hospital, Plaistow, E. D. McDonald Forbes, L.R.C.P.Edin., L.F.P.S.Glasg., appointed Deputy Medical Officer for the Eastwood District of the Basford Union. G. M. Fox, L.R.C.P.Lond., M.R.C.S.Eng., appointed Medical Officer for the Workhouse of the Walsall Union. A. Hawley, M.R.C.S.Eng., L.R.C.P.Lond., L.S.A., appointed Honorary House Surgeon to the Coventry and Warwickshire Hospital. J. A. Hedges, M.R.C.S.Eng., L.S.A., appointed Medical Officer of Health for the Eaton Bray Rural District. G. H. Hopkins, F.R.C.S.Eng., L.R.C.P.Lond., appointed Visiting Surgeon to the Lady Bowen Hospital for Women, Brisbane, Queensland. A. E. A. Lawrence, M.D.Aberd., M.B., C.M., appointed Consulting Physician Accoucheur to the Bristol General Hospital. C. A. Leedham-Green, M.D.Heidelb., F.R.C.S.Eng., appointed Surgeon for Out-patients, Queen's Hospital, Birmingham. P. R. Lowe, appointed House Physician to the Leicester Infirmary. G. H. Mapleton, M.B., C.M.Edin., appointed Medical Officer for the Goodhurst District of the Cranbrook Union. W. Millar, M.B., C.M.Glas., appointed Medical Officer and Public Vaccinator for the West Green and St. Ann's District of the Edmonton Union. W. H. C. Newnham, M.A., M.B.Camb., M.R.C S.Eng., appointed Physician Accoucheur to the Bristol General Hospital. G. S. Pope, L.R.C P., L.R.C.S.Edin., appointed Medical Superintendent of the new Asylum at Middlesbrough. R. S. McD. Pullen, appointed Surgeon to the Provident Institution of the Royal Albert Hospital, Devonport; and Honorary Anæsthetist to the South Devon and East Cornwall Hospital, Plymouth E. B. Randall, M.D.Lond., M.R.C.S.Eng., appointed Honorary Medical Officer to St. Mary's Children's Hospital, Plaistow, E. D. C. Rayner, F.R.C.S.Eng., L.R.C.P., appointed Assistant Physician Accoucheur to the Bristol General Hospital. Mr. W. G. Ridgway-Macauley, appointed Medical Officer for the Western District of the Freebridge Lynn Union. H. B. Robinson, F.R.C.S., M.D.Lond., M S., appointed Consulting Surgeon to the St. Mary's Children's Hospital, Plaistow, E. A. Tuxford, M.B.Edin., appointed Medical Officer of Health to the Rural District Council, Boston Union. E. E. Ware, M.D.Lond., B.S., M.R.C.S., appointed Honorary Medical Officer to the St. Mary's Children's Hospital, Plaistow, E. C. W. Williams, M.R.C.S , L.R.C.P.Lond., appointed Medical Officer for the Smallburgh District and the Workhouse of the Smallburgh Union. E. Young, L.R.C.P.Edin., M.R.C.S.Eng., appointed Medical Officer for the Hawkhurst District of the Cranbrook Union.

### FOREIGN MEDICAL PUBLICATIONS.

Messrs. THE SCIENTIFIC PRESS (Ltd.) beg to announce that, having made arrangements with some of the most important Continental publishers, they are now able to supply foreign medical works at the published prices.

Just Out.

**MANUEL THÉORIQUE ET PRATIQUE d'ACCOUCHMENTS.** By Dr. A. POZZI. Price 4s. 6d., net.

**PFLEGE DER HAUT.** By Dr. SPIETSCHKA and Dr. GRÜNFELD. Price 5s., net.

London: THE SCIENTIFIC PRESS (Ltd.), 28 & 29, Southampton Street, Strand, W.C.

